# Eye blink correction: a test on the preservation of common ERP components using a regression based technique

**DOI:** 10.7717/peerj.76

**Published:** 2013-05-14

**Authors:** Steven Woltering, Narges Bazargani, Zhong-Xu Liu

**Affiliations:** 1 University of Toronto, Canada; 2 University College London, England

**Keywords:** Eye blink correction, ERP, EEG, Artifacts, Go/nogo task

## Abstract

Eye blinks are a pervasive problem in electroencephalography research as they contaminate the brain signal. This paper tests the merits of a software tool employing the regression-based Gratton method that claims to remove the detrimental effects of the eye blink and leaves the activity of the brain. The efficacy of the correction tool was tested on five common stimulus-locked Event Related Potential (ERP) components used in a standard Go/Nogo task. Results suggested that the ‘corrected’ data could be predicted from data containing no eye blinks, suggesting the tool does not distort the data to great extent. This effect was found significant for all components, except for the P3. The conclusion is that this tool distorts the data at acceptable levels, yet caution should be taken when interpreting later components, like the P3.

## Introduction

Eye blinks are one of the most pervasive problems in electroencephalography (EEG) research as they contaminate brain signals and rejecting blink-trials may lead to a detrimental loss of trials and subjects. Methods attempting to mathematically correct eye blinks are often employed in Event Related Potentials (ERP) designs because, (1) ‘rejection’ can lead to an unrepresentative sample of trials, (2) the instruction to inhibit eye movements or blinks may affect task performance (Verleger, 1991), (3) some groups of subjects (children and psychiatric patients, in particular) have trouble controlling their blinking, and (4) researchers are better able to preserve trials within a reasonable experiment time, preventing issues with fatigue and boredom.

Regression-based methods (e.g., see Gratton, Coles & Donchin, 1983, for details) have been the one of the most widely used, and simple, techniques to correct for blinks to date. Such methods claim to remove the added distorting effects of the blink and leave the estimated activity of the brain. In doing so it attenuates all trials, blinks as well as no blinks. However, its merits are under debate and have mostly been evaluated using simulated data, and not directly on ERP waveforms.

The purpose of this study was to assess to what extent the Gratton regression-based correction method distorted ERP data under realistic research conditions, and with non-simulated data, in order to warrant the use of this method for studies in our lab.

In order to directly test the distorting effects of the eye blink correction method in our sample on several ERP components, we planned three main analyses:

(1) We first assessed to what extent the Gratton method, which affects all data, distorts ERP waveforms when there are no blinks. In order to do this, we will use clean trials with no blinks, and directly compared ‘corrected’ with ‘uncorrected’ trials using a within-subjects analysis.
(2) Second, we will assess how well the Gratton method corrected ERP data that had blinks. In this comparison, corrected no-blink trials were compared to corrected blink trials within each subject. Note that, in contrast to the first test, this analysis compares different trials.
(3) Third, we will test whether between-subject ERP differences could be replicated with corrected (blink and no-blink) data.

We will test the effect of the attenuation on five ERP components, namely, the N1, P2, N2, parietal P3 and the frontal P3. We feel the results of this small-scale validation study may be useful for a wider audience conducting ERP research so we wish to share them with other labs.

## Materials & Methods

### Subjects

The data from this analysis are taken from a large study investigating the neural correlates of antisocial behavior in participants, aged 7–16, from various socioeconomic backgrounds. Subjects were referred by teachers, parents or police to a clinical treatment program, which referred them to this study. A visit was booked, where the children were conducting a cognitive Go/Nogo task while an EEG was taken. Control subjects were recruited via newspaper ads.

For the current analysis, a random sample of 40 subjects was selected (20 clinicals and 20 controls). For the actual analysis, 8 participants had to be removed because they did not have enough good trials to conduct our analysis. In the end, 32 subjects were selected that had at least 10, or more, trials that contained eye blinks. The group consisted out of 17 controls (14 males) and 15 clinicals (9 males). The mean age was 10.5 years (*sd* = 2.2). For the purpose of this experiment, we were not interested in group differences and we will simply collapse across groups.

### Go/nogo task

The emotion induction go/no-go task that was used in the present study was presented using E-Prime software (Psychological Software Tools, Pittsburgh, PA). In standard Go/nogo paradigms, participants are required to press a button as fast as possible given a particular category of stimuli (the Go condition, 67%) and withhold responding given another category of stimuli (the Nogo condition, 33%). Participants in this study were instructed to click the button for each letter presented but to avoid clicking when a letter was repeated a second time in succession. A detailed description of the task can be found in Woltering et al. (2011).

### Materials & data processing

EEG was recorded using a 128-channel Geodesic Sensor Net and sampled at 250 Hz, using EGI software (Electrical Geodesics, Inc., Eugene, OR). Data acquisition was started after impedances for all EEG channels were reduced to below 50 kOhm, which is acceptable with high input impedance amplifiers (Ferree et al., 2001; Kappenman & Luck, 2010). All channels were referenced to Cz (channel 129) during recording and later rereferenced against an average reference (Tucker et al., 1993; Bertrand, Perrin & Pernier, 1985). Data were filtered off-line using a 1–30 Hz finite impulse response (FIR) bandpass. Correct no-go data were segmented into epochs from 400 ms before to 1000 ms after stimulus onset.

From this point forward, the data was split into three groups for the purposes of our analysis. To create realistic research circumstances, it’s important to note that the artifacting parameters mentioned were set a priori, without any known intention, at that time, whether this comparison was ran:

*The uncorrected no blink data*- This was ‘clean’ data, without any eyeblinks, for which no correction method was used. This data followed our standard artifacting procedure: channels were automatically marked bad when they exceeded a transition threshold of 150 µv over the entire segment (max-min). Remaining eye blinks were detected when the vertical eye channels exceeded a threshold of 150 µv (max-min) within a 160 ms (moving) time window within each trial after running a 20 ms moving-average smoothing algorithm across the entire trial period. Eye movements were detected when horizontal eye channels exceeded a threshold of 100 µv (max-min) over a 200 ms time window. Furthermore, each segment of the EEG was excluded from averaging if 20 or more channels were rejected. These settings were determined by extensive tests on a sample of the data that yielded the best artifact detection for our data. In addition, all segments were visually inspected by a trained research assistant blind to the hypotheses. After the artifacting, bad channels were removed using spherical spline interpolation.

*The corrected no blink data* – This was the same ‘clean’ data used for the uncorrected no blink data (described above), with the exception that the correction method was applied. First, the data followed the exact same artifacting procedure as described above, after which bad channel replacement was run. Then, the eye blink correction tool was run (setting the blink slope threshold at the recommended 12 µV/ms), followed by another, somewhat stricter, round of artifacting and subsequent bad channel replacement. This round of artifacting was mostly meant to remove anomalies in the data that were leftover by the correction data. This amounted to the rejection of less than 5% of the total trials.

*The corrected blink data*- This data contained eye blinks that were corrected using the above-mentioned procedure using the Gratton correction algorithm. These were data from the same participants used in the no blink data.

After these procedures, data were averaged, average referenced, coded, and baseline corrected for the 400 ms preceding the stimulus onset. The coding was performed by research assistants blind to the hypothesis of the study. Five common stimulus-locked ERP components, such as the N1, P2, N2, P3p (parietal P3), and the frontal P3, were derived when subjects correctly inhibited on a nogo trial using the traditional peak picking method. The N1, P2, and N2 were coded as maximum positivities or negativities before 500 ms after the stimulus had appeared, whereas the P3p and P3 were typically coded 500 ms post stimulus (to max of 900 ms). Data were analyzed according to standard lab procedures (for more details, see Woltering et al., 2011) using IBM SPSS statistics software (version 20). Outliers were removed if they were two standard deviations above the mean. In the current analyses, no more than 3 outliers were removed in total.

## Results

**Figure 1:**
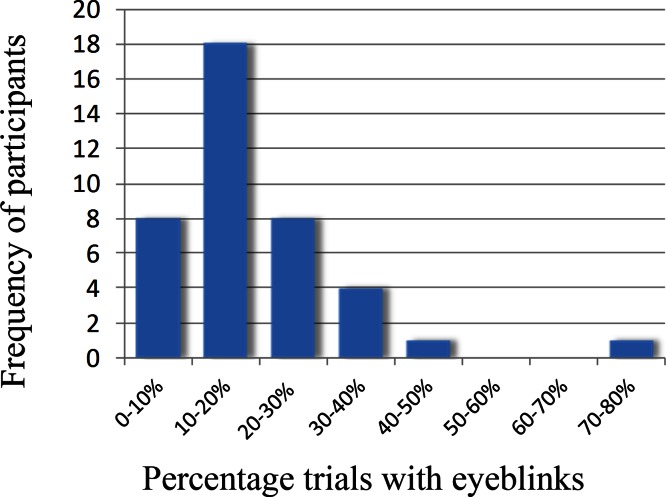
Frequency plot showing the number of participants and the percentage of eyeblinks in the trials.

We first determined to what extent our initial sample of 40 participants suffered from eyeblinks in our paradigm. Indeed, an average of 19% of the trials contained eye-blinks. Figure 1 shows the frequency of participants and what percentage of trials contained eyeblinks.

*Analysis #1: Since the Gratton correction method affects all data, to what extent does it distort the data when there are no blinks?*

**Figure 2:**
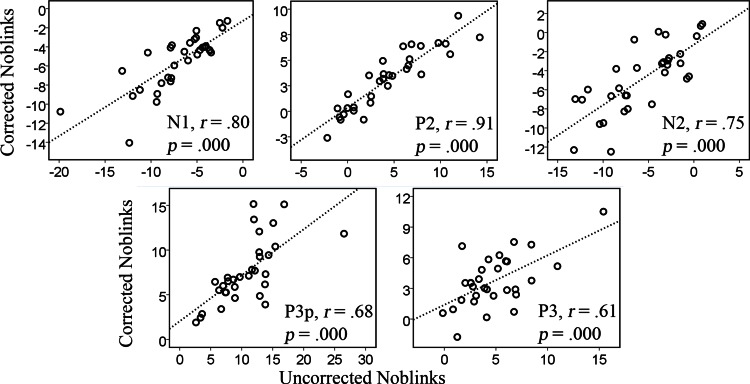
Correlations between the uncorrected and corrected data containing no blinks for the amplitudes of the various components, N1, P2, N2, P3p, P3.

Results from the simple linear regression analyses in Table 1 show that the correction tool does not distort the data to great extent and that corrected data could be predicted from the original data. Figure 2 and Table 1 show that the predictive relations are strong and linear.

**Table 1:** Simple linear regression results for predicting corrected no-blink amplitudes from uncorrected no-blink amplitudes for 5 different ERP components.

Component	Standardized beta coefficient	Regression model fit (ANOVA)	*p*-value (Model)
N1	.803	*F*(1, 29) = 52.50	.000
P2	.918	*F*(1, 29) = 155.95	.000
N2	.764	*F*(1, 29) = 40.72	.000
P3p	.685	*F*(1, 29) = 25.65	.000
P3	.607	*F*(1, 29) = 16.93	.000

The results seem stronger for the early components, like the N1 and P2, which are generated in posterior regions of the brain and happen at an earlier stage. The *R*^2^ values for the P3 component, however, were not very high, *R*^2^ = .21. The earlier components have *R*^2^ values of .72 (N1), .87 (P2), .64 (N2), and .51 (P3p), explaining more than 50% of the variance. The correlations for the first analysis are plotted in Fig. 2 and show a similar pattern whereby the later components show somewhat weaker correlations than the earlier ones.

As Table 2 shows, the main amplitudes are less for the corrected data. However, this seems to be a systematic effect and due to subtraction of the eye-electrodes in the algorithm of the correction tool. Paired sample t-tests revealed that this difference is significant in all cases, except for the N2 component at the level of a trend (*p* = .10).

**Table 2:** Mean amplitudes and standard deviations of the uncorrected and corrected data containing no eye blinks for 5 different ERP components.

Component	Uncorrected mean (*sd*)	Corrected mean (*sd*)
N1	−7.07 (3.84)	−5.54 (2.90)
P2	4.35 (4.21)	3.12 (2.92)
N2	−5.65 (4.18)	−4.84 (3.51)
P3p	10.67 (4.72)	7.53 (3.54)
P3	5.32 (4.33)	3.69 (2.5)

*Analysis #2: How well does the Gratton method correct data that had blinks?*

The second analysis, testing whether the corrected blink data could be compared to the corrected noblink data, showed a similar pattern. Table 3 shows the results for the regression analyses. The parameters were significant for the different components at a .05 level, except for the P3 component. The analysis was expected to fit less well because the data compared are also derived from different trials, but also because the tool’s correction was really put to the test. The model for the N1, P2, and N2 components each predicted more than 50% of the variance (*R*^2^ = .52, .60, .60, respectively), whereas models for the later components, like the P3p and the P3 predicted 45% and 23%, respectively.

**Table 3:** Simple linear regression results for predicting corrected blink amplitudes from corrected no-blink amplitudes for 5 different ERP components.

Component	Standardized beta coefficient	Regression model fit (ANOVA)	*p*-value (Model)
N1	.587	*F*(1, 29) = 15.22	.001
P2	.793	*F*(1, 29) = 48.98	.000
N2	.753	*F*(1, 29) = 37.92	.000
P3p	.627	*F*(1, 29) = 18.76	.000
P3	.267	*F*(1, 29) = 2.23	.146

**Figure 3:**
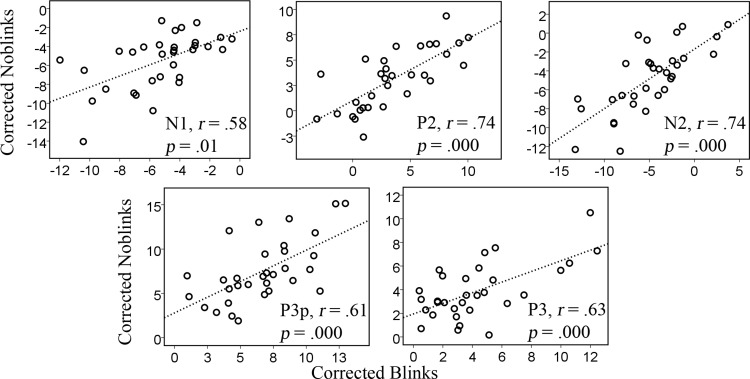
Correlations between corrected data previously containing blinks and no-blinks for the amplitudes of the various components, N1, P2, N2, P3p, and P3.

The correlations were relatively large, but, as expected, not as strong as those of the first analysis. In general, the linear relation is seen in all cases in Fig. 3.

Table 4 shows that the differences in absolute amplitude were similar. Paired sample t-tests revealed that none of these were significantly different.

**Table 4:** Mean amplitudes and standard deviations of the corrected data comparing blink trials with noblink trials for 5 different ERP components.

Component	Blink mean (*sd*)	Noblink mean (*sd*)
N1	−5.47 (2.76)	−5.62 (2.91)
P2	3.44 (3.58)	3.16 (2.95)
N2	−5.04 (4.18)	−4.80 (3.57)
P3p	6.56 (3.04)	7.48 (3.59)
P3	4.27 (3.27)	3.86 (2.34)

**Figure 4:**
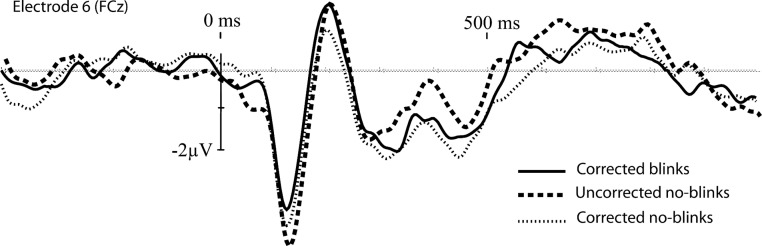
Baseline corrected grand-average waveforms for the corrected blinks, and the uncorrected and corrected noblinks, for electrode site FCz.

**Figure 5:**
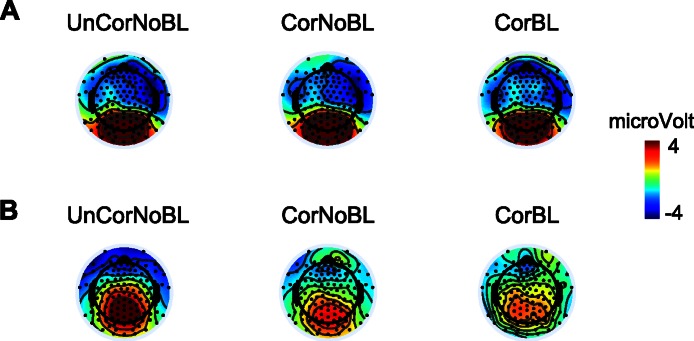
Topoplots showing activation between different conditions and time windows. Topoplots of an early (A) time window (100–200 ms) and late (B) time window (500–600 ms) between the different conditions: UnCorNobL = Uncorrected Noblinks; CorNoBL = Corrected Noblinks; CorBL = Corrected Blinks.

Grand average waveforms from electrode site 6 (one of the frontal sites, and primary location to detect the N2) are plotted in Fig. 4 and provide a general impression of the correction tool’s performance. The corrected blink data looks similar to the corrected noblinks and uncorrected noblinks. Figure 5 shows topoplots comparing activation from all 128 channels between our uncorrected noblink, corrected noblink, and corrected blink conditions for an early and late time window. These data show relatively minor distortion, with fewer distortions for the early time window.

*Analysis #3: Can ‘traditional’ between subject ERP differences be replicated with corrected (blink and no-blink) data?*

Uncorrected no-blink ERP differences were found in two analyses (i.e., gender and age). Patterns were compared using corrected blink trials and corrected no-blink trials.

Results show that, generally, patterns remain consistent in magnitude, direction and significance for gender differences that were found, as well as correlations with age. Some deviations were found for the later components, such as the P3 (see Table 5).

**Table 5:** Analyses showing how similar outcomes are between (un)corrected noblink and corrected blink data. Analysis (A) shows gender differences, as tested by independent sample t-tests, for each of the ERP compoments for each condition. Analysis (B) shows the Pearson correlations between age and the various ERP components for each condition.

Analysis A: Gender * ERP (Group difference *t*-test)				Analysis B: Age * ERP (Pearson correlation)		
ERP	Uncorrected noblinks	Corrected noblinks	Corrected blinks	Uncorrected noblinks	Corrected noblinks	Corrected blinks
N1	*p* = .373	*p* = .153	*p* = .124	.45^*^, *p* = .01	.52^**^, *p* = .00	.37^*^, *p* = .04
P2	*p* = .078	*p* = .153	*p* = .146	−.33, *p* = .07	−.29, *p* = .11	−.21, *p* = .27
N2	*p* = .034^*^	*p* = .007^**^	*p* = .038^*^	.12, *p* = .53	.27, *p* = .14	.31, *p* = .10
P3p	*p* = .233	*p* = .080	*p* = .040^*^	−.40^*^, *p* = .03	−.46^*^, *p* = .01	−.39^*^, *p* = .03
P3	*p* = .830	*p* = .272	*p* = .872	−.14, *p* = .44	−.41^*^, *p* = .02	−.11, *p* = .56

**Notes.**

* *p* < .05.

** *p* < .01.

## Discussion

The results from our first analysis showed that the corrected data could be predicted from the uncorrected (i.e., original) data. Since it is inherent to the tools algorithm that it will perform a manipulation on the good data (containing no eye blinks), the level of distortion needed to be tested. The results strongly suggest that the tool does not distort ‘good’ data for any component and that it is safe to use in that respect. Differences were found in absolute amplitudes; however, these were largely systematic across the board and due to the subtraction method of the correction tool. Since this tool will be applied to data used for within and between subjects analysis in a standardized fashion, these differences, if systematic, will not pose any problems to the interpretation of data.

The second analysis investigated the level to which the correction distorted the data when correcting eye blinks. Corrected blink data could be predicted from all the corrected noblink data, however, the later components (i.e., P3p, P3) showed more difficulty being predicted whereby the P3 could not be predicted at all. It is possible that the P3, being a slower component that is spread out across more time, could be more prone to error compared to more defined, and faster, components such as the P2. Interestingly, no pattern was found for location. It could be conceived that frontal components, being closer to the eyes, would be more susceptible to any potential distortive effects. A frontal component, such as the N2, however, did not show any distortion and even showed more consistent correlations than the stable early components (i.e., N1, P2).

The third analysis investigated whether statistical analyses as they would normally be conducted could be replicated with corrected data with blinks and no blinks. Note that the latter data set consists out of data that would normally be rejected. The results showed that patterns in the data generally remained, with the exception of the P3, lending credence to the notion that the tool corrects trials that would otherwise be rejected.

## Conclusions

To conclude, considering the amount of trials and subjects lost due to eye blinks in developmental neuroscience using psychiatric populations, the Gratton methods seems to correct blinks with minimal levels of distortion considering uncorrected blink data would otherwise be thrown out. Caution should be taken, however, when interpreting later components, such as the P3, which seem more sensitive to distortions. This would make the tool useful, in particular, for the investigation of early perceptual and cognitive processes, such as the attentional modulation of early components occurring before 100 ms (Gazzaley, 2011) and conflict monitoring processes reflected by the N2 (Donkers & van Boxtel, 2004; Huster et al., 2012). A limitation to bear in mind is that the current analysis was performed under certain experimental circumstances (i.e., a *go-nogo* task) and these results would not necessarily generalize to ERP research in general. Compared to other more sophisticated correction methods, the Gratton method requires less data manipulation, computation, and time, which offers considerable advantage. Studies directly comparing eye-blink (not eye movement) correction methodologies suggest regression-based methods are not inferior to ICA and source dipole methods (Hoffmann & Falkenstein, 2008; Lins et al., 1993; Wallstrom et al., 2004). We would therefore recommend this method to be applied for cognitive and visuo-perceptual research investigating relatively early ERP components.
